# Clinical Validation of Patient-Reported Cognitive Assessment Tools for Adverse Effects of Electroconvulsive Therapy in Patients with Unipolar Depression

**DOI:** 10.1192/j.eurpsy.2026.11753

**Published:** 2026-07-31

**Authors:** A. B. Dall, E. Salagre, A. L. M. Carmo, M. Sørensen, R. Laursen, A. B. Mathiassen, S. D. Østergaard, P. Kølbæk

**Affiliations:** 1 Department of Affective Disorders, Aarhus University Hospital - Psychiatry, Aarhus; 2 Center for Neuropsychiatric Depression Research, Mental Health Center Glostrup, Copenhagen University Hospital - Mental Health Services CPH, Glostrup; 3 Department of Clinical Medicine, University of Copenhagen, Copenhagen; 4 Department of Clinical Medicine, Aarhus University, Aarhus, Denmark

## Abstract

**Introduction:**

Electroconvulsive therapy (ECT) is highly effective for several severe psychiatric conditions, but its cognitive adverse effects remain a key concern and are challenging to monitor with resource-intensive objective tests.

**Objectives:**

This prospective observational study aims to validate two patient-reported outcome measures (PROMs) for assessing cognitive adverse effects in patients with unipolar depression undergoing ECT. The psychometric properties of the PROMs will be assessed against several clinician-administered scales.

**Methods:**

Participants will complete the Squire Subjective Memory Questionnaire (SSMQ) and the Global Self-Evaluation of Memory (GSE-My) at baseline, after the sixth ECT session, and at the end of the treatment course. Clinician-administered assessments will include the Screen for Cognitive Impairment in Psychiatry (SCIP) and the Columbia Autobiographical Memory Interview Short-Form (CAMI-SF), along with measures of depressive symptom severity, functional impairment, and global clinical impression. Psychometric evaluation will focus on test–retest reliability, construct validity, and responsiveness of the PROMs compared with the clinician-rated scales.

**Results:**

Enrollment and data collection are expected to begin at the end of September 2025. Preliminary findings will be presented at the 34^th^ European Congress of Psychiatry in March 2026.

**Conclusions:**

If a patient-reported scale is found to have acceptable psychometric properties, this could be a highly feasible method of monitoring cognitive adverse effects of ECT in clinical practice, thereby supporting clinicians in making informed, timely adjustments to treatment in order to minimize these effects.

**Disclosure of Interest:**

None Declared

